# Effects of six different exercise therapies on sleep indices, cardiopulmonary endurance, and body composition in patients with sleep-related breathing disorders: network meta-analysis of 40 RCTs

**DOI:** 10.3389/fphys.2026.1773901

**Published:** 2026-03-25

**Authors:** Zhenhao Ma, Qinghui Li

**Affiliations:** 1 Zhejiang Business Technology Institute, Zhejiang, China; 2 Zhejiang Fashion Institute of Technology, Zhejiang, China

**Keywords:** body composition, cardiopulmonary endurance, exercise therapy, sleep apnea syndrome, sleep quality

## Abstract

**Background:**

Sleep apnea syndrome is a common sleep disorder that frequently leads to multi-systemic damage. Traditional therapies have limitations, while exercise interventions show potential; however, the efficacy of different exercise modalities remains unclear.

**Methods:**

Six databases (PubMed, Web of Science, EBSCO, Embase, Cochrane Library, and China National Knowledge Infrastructure) were systematically searched. A total of 40 randomized controlled trials involving 1790 patients with Sleep Apnea Syndrome were included. The outcomes comprised key indexes for evaluating sleep indices, cardiorespiratory fitness, and body composition. A network meta-analysis was conducted using Stata 18.0 to assess the relative effectiveness of each exercise intervention and to evaluate the consistency between direct and indirect evidence.

**Results:**

Network meta-analysis demonstrated that High-Intensity Interval Training (HIIT) was the most effective intervention for improving sleep indices (SUCRA = 98.9, PrBest=95.1%) and cardiorespiratory fitness (SUCRA = 87.9, PrBest=67.8%). Aerobic Training (AT) ranked highest for improving body composition (SUCRA = 92.7, PrBest=71.4%). Significant heterogeneity was observed in several comparisons, particularly regarding body composition outcomes. Direct comparisons revealed that Aerobic Combined with Resistance Training (ACRT) was significantly more effective than Respiratory Exercise (RE) in improving sleep indices (SMD = -2.97, 95% CI [-4.11, -1.82], P < 0.0001).

**Conclusion:**

This analysis indicates that exercise interventions differentially improve outcomes in SAS. HIIT appears optimal for sleep and cardiorespiratory benefits, while AT best targets body composition. Clinical application should be personalized based on patient-specific treatment priorities.

**Systematic Review Registration:**

https://www.crd.york.ac.uk/prospero/, identifier CRD420251253035.

## 1 Introduction

Sleep Apnea Syndrome (SAS) is a prevalent and serious sleep disorder characterized by recurrent episodes of partial or complete collapse of the upper airway during sleep (McEvoy et al., 2016). The primary diagnostic metric, the Apnea-Hypopnea Index (AHI), quantifies the severity of these respiratory disturbances. With a global adult prevalence estimated between 9% and 38%, SAS represents a significant public health burden, strongly associated with obesity and aging demographics (Senaratna et al., 2017).

The pathophysiological consequences of SAS extend beyond nocturnal symptoms, conferring substantial multi-system morbidity and increased mortality risk (Malhotra et al., 2024). Chronic intermittent hypoxia and sleep fragmentation drive systemic inflammation, oxidative stress, and autonomic dysfunction, establishing SAS as an independent risk factor for cardiovascular diseases, metabolic disorders, and neurocognitive impairment (Javaheri et al., 2017). This analysis focuses on three critical and interrelated outcome domains central to SAS management. First, sleep indices (SI, e.g., AHI, oxygen saturation, sleep architecture) directly measure disorder severity. Second, diminished cardiorespiratory fitness (CRF), reflected by reduced peak oxygen consumption, is a hallmark of SAS and a key predictor of cardiovascular health (Reutrakul and Van Cauter, 2018). Third, adverse body composition (BC), particularly increased visceral and upper-body adiposity, is both a cause and consequence of SAS, exacerbating airway collapsibility and metabolic dysfunction (Vranish and Bailey, 2016).

Traditional management of moderate-to-severe SAS primarily relies on device-based therapies such as Continuous Positive Airway Pressure (CPAP), which is highly effective but suffers from variable long-term adherence due to side effects and comfort issues (Schwartz et al., 2008). Surgical options, while beneficial for select patients, are invasive and carry inherent risks. Crucially, these conventional approaches primarily address the mechanical obstruction without directly targeting the underlying systemic deficits in CRF and BC (Patil et al., 2019). This limitation has spurred interest in exercise therapy as a fundamental complementary strategy. Evidence suggests that structured exercise can reduce SAS severity through multiple mechanisms, including strengthening upper airway muscles, improving autonomic regulation, and reducing inflammation (Weaver and Grunstein, 2008). Importantly, exercise simultaneously targets all three core domains: improving sleep parameters, enhancing CRF, and promoting favorable BC changes.

The therapeutic application of exercise in SAS encompasses diverse modalities. This meta-analysis will specifically evaluate and compare six distinct interventions: High-Intensity Interval Training (HIIT), Circuit Training (CT), Aerobic Training (AT), Respiratory Exercise (RE), Aerobic Combined with Resistance Training (ACRT), and Mind-Body Exercise (MBE). HIIT offers time-efficient improvements in cardiovascular and metabolic health. CT provides combined strength and aerobic conditioning. AT forms the foundation for enhancing endurance and oxygen utilization. RE directly targets respiratory muscle function. ACRT integrates the benefits of both aerobic and resistance training. MBE emphasizes the mind-body connection to improve autonomic regulation and stress response. Each modality presents a unique physiological approach to interrupting the SAS pathological cycle (Iftikhar et al., 2014).

In summary, while the detrimental multi-systemic impacts of SAS are well-established and the limitations of conventional therapies like CPAP are recognized, exercise therapy has emerged as a promising adjunctive strategy. Despite the growing body of evidence supporting various exercise modalities, a critical evidence gap remains: the comparative effectiveness of different exercise types on the core SAS outcomes of sleep quality, cardiorespiratory fitness, and body composition has not been systematically quantified. Therefore, the primary objective of this study is to conduct a network meta-analysis of randomized controlled trials to compare the relative efficacy of six distinct exercise therapies—High-Intensity Interval Training, Circuit Training, Aerobic Training, Respiratory Exercise, Aerobic Combined with Resistance Training, and Mind-Body Exercise—in patients with Sleep Apnea Syndrome. This study aims to provide a hierarchical ranking of these interventions, offering crucial insights to inform personalized, evidence-based exercise prescriptions. By identifying the most effective modalities for specific clinical goals, this research seeks to optimize the non-pharmacological management of SAS, ultimately improving patient-centered outcomes and filling a critical void in current clinical guidelines.

## 2 Methods

This study adheres to the Preferred Reporting Items for Systematic Reviews and Meta-Analyses (PRISMA) guidelines and the Cochrane Handbook for Systematic Reviews of Interventions.

Registration number: **CRD420251253035**.

### 2.1 Data sources and retrieval strategy

Two researchers independently conducted systematic searches of the PubMed database. The search period spanned from database inception to May 2024, limited to English-language human studies. The search strategy employed a combination of Medical Subject Headings (MeSH) and free-text terms, which underwent pre-testing and iterative refinement to ensure both recall and precision. For Sleep Apnea Syndrome, MeSH terms included: “Sleep Apnea Syndromes”[Mesh], “Sleep Apnea, Obstructive”[Mesh], with free-text terms such as “sleep apnoea,” “OSA,” and “SAS.” For exercise therapy, MeSH terms included: “Exercise”[Mesh], “Exercise Therapy”[Mesh], with free-text terms like “physical activity,” “training,” and “rehabilitation.” Specific free-text terms for the six interventions included: “high-intensity interval training,” “HIIT,” “circuit training,” “aerobic training,” “respiratory exercise,” “breathing exercise,” “aerobic combined resistance training,” “mind-body exercise,” “yoga,” and “tai chi.” These concept groups were combined using Boolean operators (AND, OR, NOT). Concurrently, the reference lists of included studies and relevant review articles were manually screened to prevent literature omission.

### 2.2 Study selection and inclusion criteria

#### 2.2.1 Study inclusion criteria were formulated based on the PICOS principle

##### 2.2.1.1 P (population)

Adult patients aged ≥18 years diagnosed with sleep apnoea syndrome (SAS) via polysomnography (PSG) or home sleep apnoea testing, typically based on an apnoea-hypopnoea index (AHI) ≥5 events per hour. The study did not restrict participants based on SAS subtype (predominantly obstructive), disease severity, duration of illness, or use of baseline treatments such as continuous positive airway pressure (CPAP). However, these factors were considered in analyses or used as criteria for subgroup analyses. Participants with concomitant severe respiratory diseases, unstable cardiovascular conditions, or musculoskeletal disorders limiting exercise capacity—which could compromise exercise intervention safety or outcome assessment—were excluded.

##### 2.2.1.2 I (intervention)

The study intervention shall comprise one of the following six non-pharmacological, non-invasive exercise therapies, delivered as a structured, planned, supervised, or explicitly guided training programme: High-Intensity Interval Training (HIIT), Circuit Training (CT), Aerobic Training (AT), Respiratory Exercise (RE), Aerobic Combined with Resistance Training (ACRT), Mind-Body Exercise (MBE).

The control group is defined as: no specific exercise intervention (e.g., usual care, waiting list, health education), or other types of low-intensity/placebo exercise.

##### 2.2.1.3 C (control measures)

Usual care, sham exercise intervention, health education, or cross-comparisons between different types of the six exercise therapies.

##### 2.2.1.4 O (outcome measures)

Studies must report quantitative data for at least one core outcome dimension:

1. Sleep indices: Primarily including AHI (Apnoea-Hypopnoea Index), ESS (Epworth Sleepiness Scale), TST (Total Sleep Time), Sleep efficiency, Arousal index, ODI (Oxygen Desaturation Index).
2. Cardiopulmonary endurance: Key indicators include SBP (Systolic Blood Pressure), DBP (Diastolic Blood Pressure), Lowest SaO_2_(Lowest Oxygen Saturation), Mean SaO_2_(Mean Oxygen Saturation), FVC (Forced Vital Capacity), FEV_1_ (Forced Expiratory Volume in 1 second), VO2 peak (Peak Oxygen Consumption), PEF (Peak Expiratory Flow).
3. Body composition: Primary indicators include BMI (Body Mass Index), Neck circumference, Weight, Waist circumference, Total cholesterol, Glucose, HDL (High-Density Lipoprotein Cholesterol), LDL (Low-Density Lipoprotein Cholesterol), Triglycerides.

##### 2.2.1.5 S (study type)

Randomised controlled trials (RCTs), including parallel or crossover designs. Language restricted to Chinese and English.

To ensure transparency and reproducibility, the six exercise interventions were operationally defined based on common protocols identified in the literature. While specific parameters varied across the included randomized controlled trials, the interventions were categorized according to the following general methodological characteristics:

High-Intensity Interval Training (HIIT): Interventions involved repeated bouts of high-intensity exercise (typically ≥80% of maximum heart rate or peak oxygen uptake) interspersed with periods of low-intensity recovery or rest.

Circuit Training (CT): This modality consisted of a series of exercises performed in rotation with minimal rest between stations, combining both resistance and aerobic elements to maintain an elevated heart rate.

Aerobic Training (AT): Interventions were characterized by continuous, rhythmic, and moderate-intensity activities (e.g., walking, cycling, jogging) aimed at improving cardiorespiratory endurance.

Respiratory Exercise (RE): This category encompassed interventions specifically targeting the respiratory muscles, including inspiratory muscle training (IMT) using threshold devices, expiratory muscle strength training (EMST), and oropharyngeal exercises.

Aerobic Combined with Resistance Training (ACRT): These programs integrated both aerobic and resistance training components within a single session or on alternating days, targeting both cardiorespiratory and muscular fitness.

Mind-Body Exercise (MBE): Interventions such as yoga and Tai Chi were included in this category, which emphasize the integration of physical postures, breathing control, and meditation to improve mental and physical well-being.

#### 2.2.2 Exclusion criteria

1. Studies involving SAS patients with other severe comorbidities (e.g., advanced heart failure, severe chronic obstructive pulmonary disease) that may dominate functional outcomes.
2. Interventions involving combined therapies (e.g., exercise combined with surgery/medication) where data isolating the effect of exercise alone cannot be extracted.
3. Non-randomised controlled trial designs, including non-randomised controlled studies, single-group pre-post studies, observational studies, reviews, systematic reviews, case reports, and conference abstracts.
4. Animal studies.
5. Studies where full-text access was unavailable, or where full-text data were missing, inaccessible, or could not be obtained despite contacting the original authors.
6. Duplicated publications; only the most comprehensive or most recent version would be included.

### 2.3 Data extraction

Literature screening and data extraction were conducted independently by two researchers. First, duplicate records were removed from all retrieved literature using EndNote X9 software. Subsequently, preliminary screening was performed by reviewing titles and abstracts, followed by full-text reading of remaining studies to determine final inclusion. The two researchers cross-checked their screening results (M.Z and L.Q). Disagreements were resolved through mutual discussion or consultation with a third researcher (C.X). Data extraction employed a pre-designed standardised form, capturing: - Study basic information (first author, publication year, country) - Participant characteristics (sample size per group, age, gender, body mass index, diagnostic criteria and baseline severity of sleep apnoea syndrome) - Detailed intervention measures (specific exercise therapy type, intervention frequency, session duration, total treatment duration, exercise intensity, and protocol description) - Outcome measure data (primarily covering three domains: SI, cardiopulmonary endurance, and BC) detailed intervention measures (specific exercise therapy type, intervention frequency, single session duration, total treatment duration, exercise intensity, and specific protocol description), and outcome measure data (primarily mean, standard deviation, or change values for specific indicators across three domains: SI, cardiorespiratory endurance, and BC, before and after treatment). Where studies included multiple follow-up time points, assessment data from the most recent post-intervention evaluation were extracted for primary analysis. For studies with incomplete data reporting, we shall contact the original corresponding author via email to obtain necessary information. Should no response be received within two weeks, a reminder email shall be sent. If complete data remains unavailable, the study shall be excluded from quantitative analysis.

### 2.4 Risk of bias in included studies

Two researchers independently assessed risk of bias using the Cochrane 5.1 risk of bias tool across seven domains: random sequence generation; allocation concealment; blinding of patients and therapists; blinding of outcome assessors; incomplete outcome data; selective reporting of results; and other sources of bias. Subsequently, Review Manager 5.3 was employed to analyse the risk assessments, categorising each study domain as “low risk”, “high risk”, or “unclear”. Finally, the two researchers cross-checked their respective findings; any discrepancies were resolved through mutual discussion or consultation with a third researcher to determine the final inclusion status of studies.

### 2.5 Statistical analysis

This study employed Stata 18.0 software to conduct a network meta-analysis, evaluating the effects of six exercise therapies (HIIT, CT, AT, RE, ACRT, MBE) on SI, cardiopulmonary endurance (CE), and BC in patients with sleep apnoea syndrome. All continuous outcome measures were expressed as standardised mean differences with 95% confidence intervals as effect sizes. A random-effects model was employed to pool effect sizes, accounting for inter-study heterogeneity assessed via the I² statistic and Cochran’s Q test. Network relationship diagrams visualised comparative relationships between interventions, whilst contribution plots analysed each direct comparison’s contribution to network estimates. Corrected comparison funnel plots assessed publication bias, and the area under cumulative ranking probability curves ranked the relative efficacy of the six exercise interventions.

To assess the consistency between direct and indirect evidence within the network, we conducted both local and global inconsistency tests. Local inconsistency was evaluated using the node-splitting method, which separates evidence for a specific comparison into direct and indirect components and tests for disagreement between them. A P-value less than 0.05 was considered indicative of significant inconsistency. Additionally, global inconsistency was assessed using the design-by-treatment interaction model to examine the overall coherence of the network. These analyses were performed using the network sides and network meta inconsistency commands in Stata 18.0. No significant inconsistency was detected (all P > 0.05), supporting the validity of the consistency assumption underlying the network meta-analysis. The surface under the cumulative ranking (SUCRA) curve was calculated to provide a numerical summary of the probability that each intervention is among the best options. SUCRA values range from 0% to 100%, where a value closer to 100% indicates a higher likelihood that the intervention is among the most effective, whereas a value near 0% suggests the intervention is likely among the least effective. These rankings allow for a hierarchical comparison of the six exercise interventions across different outcome domains.

## 3 Results

### 3.1 Literature screening results

**Figure 1:**
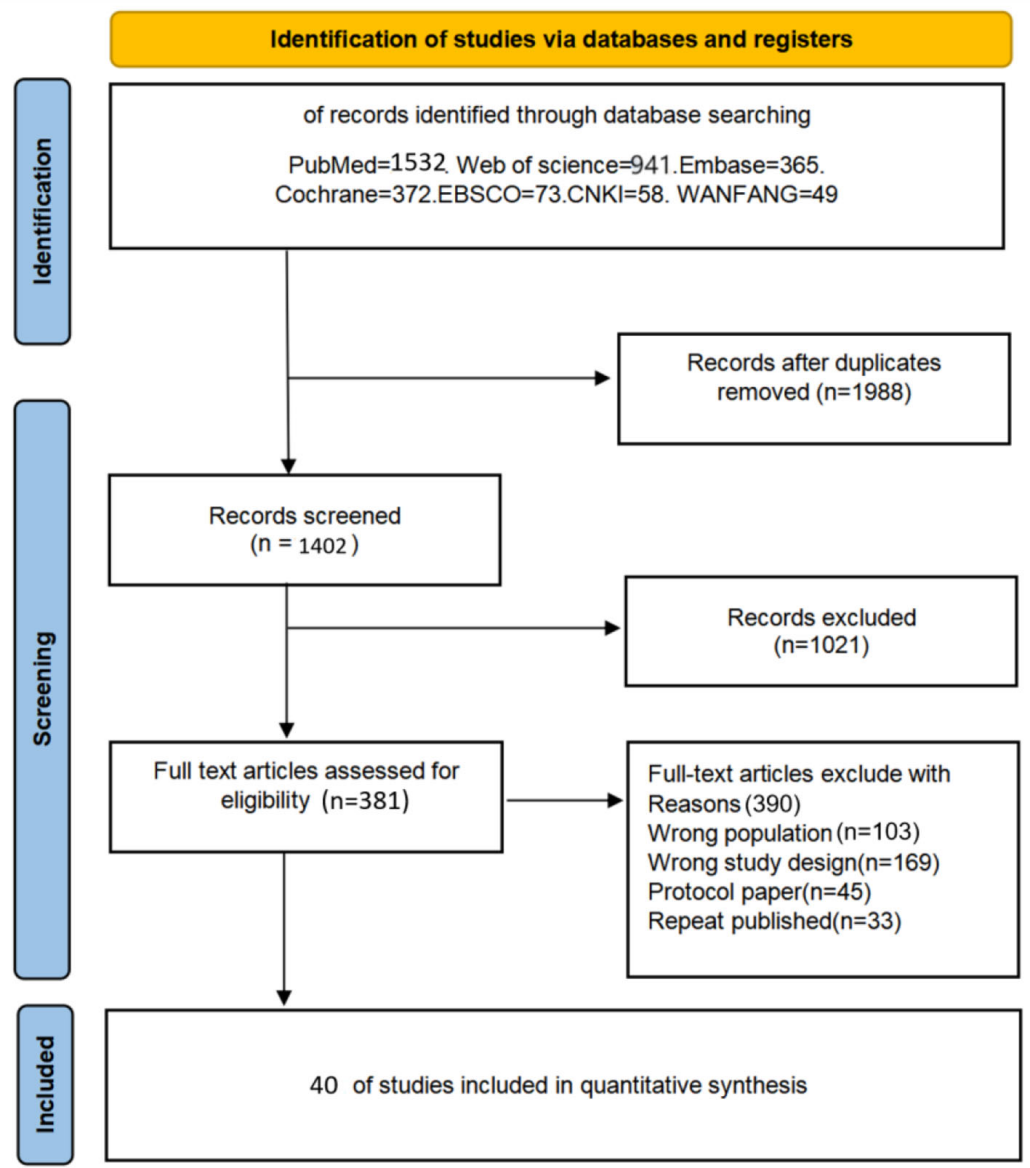
Literature search flowchart.

The literature screening flowchart is presented in **Figure 1** A total of 3390 potentially eligible studies were retrieved from various databases. To ensure study accuracy and prevent duplicate counting of identical content, 1988 duplicate records were excluded through automated and manual checks, leaving 1402 studies for screening. By analysing the titles and abstracts of each study, 1021 studies failing to meet the inclusion criteria were excluded, retaining only those most relevant to the research objectives. Full texts of 390 articles were obtained and reviewed, with detailed assessment of their study design, sample size, methodological quality, and research outcomes. This process ultimately identified 40 randomised controlled trials meeting the study’s quality criteria, evaluating six distinct forms of rehabilitation intervention. Each step of the screening process adhered to a strictly standardized protocol to ensure the reliability and scientific validity of the results.

### 3.2 Basic characteristics of included studies

The basic characteristics of the 40 included studies are detailed in **Table 1**. These studies were published between 2009 and 2024, conducted across multiple countries including Turkey, China, India, the UK, Brazil, France, the USA, Canada, Spain, Japan, Egypt, Switzerland, Thailand, and Italy, involving a total of 1,790 patients with SAS. Regarding interventions, the network encompassed all six predefined exercise therapies. Treatment durations ranged from 4 to 40 weeks.

**Table 1:** Basic characteristics of included studies.

Study	Country	Group	Sample (M/F)	Age (mean ± SD)	Intervention	Intervention frequency and duration	Duration	AHI	ESS
Yilmaz Gokmen 2018 (Yilmaz Gokmen et al., 2019)	Turkey	MBTR	25 (13/12)	50.44–8.38	TC+FMT	1h, 5times/week	12w	19.32 ± 7.09	9.56 ± 5.68
		CON	25 (18/7)	45.68–7.64	Home-based exercise	1h, 5times/week		18.66 ± 6.14	8.20 ± 5.25
Pei 2022 (Pei et al., 2022)	China	MBTR	20 (20/0)	20.8 ± 1.3	BTG	10min, 2times/day	12w		10.15 ± 1.57
		CON	20 (20/0)	20.7 ± 1.1	CON	/			10.59 ± 1.52
Gupta 2023 (Gupta et al., 2023)	India	MBTR	18 (12/6)	46.2 ± 9.4	CON	100min, ≥5days/week	12w	51.2 ± 28.0	11 ± 5.9
		CON	19 (15/4)	45.7 ± 10.7	OSA yoga	60min, ≥5days/week		47.2 ± 23.0	11.6 ± 6.3
Moss 2014 (Moss et al., 2014)	UK	HIITR	30	18-85	IG	1h, 3days/week	12w		
		CON	30		CON	/			
da Silva 2022 (Da Silva et al., 2022)	Brazil	HIITR	12 (7/5)	71 ± 5.3	Resistance Training Group	1h, 2days/week	12w	30 ± 7	
		CON	11 (6/5)	72 ± 5.0	IET	/		29 ± 9	
Desplan 2014 (Desplan et al., 2014)	France	CTR	11	35-70		2h, 6days/week	4w	40.6 ± 19.4	13.6 ± 4.5
		CON	11		SOHE	2days/week		39.8 ± 19.2	8.0 ± 5.7
Bughin 2020 (Bughin et al., 2020)	France	CTR	34 (25/9)	53.71 ± 7.30	IET+EC	120min, 3days/week	8w	28.15 ± 9.55	
		CON	34 (30/4)	55.00 ± 7.61	EC	30-45min, 1day/week		26.10 ± 11.69	
Araujo 2021 (Araújo et al., 2021)	Brazil	CTR	16 (6/10)	50 ± 6	ET	60min, 3days/week	40 ± 3.9w	44 ± 31	
		CON	18 (12/6)	54 ± 8	Untraine	/		45 ± 27	
Goya 2021 (Goya et al., 2021)	Brazil	CTR	18 (9/9)	53.8 ± 1.7	EG	60min, 3days/week	40 ± 3.9w	45.8 ± 7.8	11.3 ± 1.4
		CON	15 (10/5)	49.3 ± 1.7	CON	/		39.8 ± 5.5	12.7 ± 1.4
Kline 2011 (Kline et al., 2011)	USA	ARTR	27 (15/12)	47.6 ± 1.3	EG	40min, 4days/week	12w	32.2 ± 5.6	
		CON	16 (9/7)	45.9 ± 2.2	SCG	15min, 2days/week		24.4 ± 5.6	
Kline 2012 (Kline et al., 2012)	USA	ARTR	27 (15/12)	47.6 ± 1.3	EG	40min, 4days/week	12w	32.2 ± 5.6	
		CON	16 (9/7)	45.9 ± 2.2	SCG	15min, 2days/week		24.4 ± 5.6	
Berger 2018 (Berger et al., 2018)	France	ARTR	43		EG	60min, 3days/week	36w	23.1 ± 8.0	8.4 ± 4.4
		CON	45		CON	/		20.7 ± 6.1	7.4 ± 4.3
Servantes 2018 (Servantes et al., 2018)	Brazil	RBAECWPAP	17 (12/5)	53 ± 10	Exercise+CPAP	≥4h, 7days/week	12w	25 ± 15	9 ± 5
		ARTR	18 (8/10)	51 ± 9	Exercise	30min, 3days/week (first month); 45min, 3days/week (second month)		28 ± 17	11 ± 3
Berger 2019 (Berger et al., 2019)	France	ARTR	36 (24/12)	62 ± 5.91	EG	1h, 3days/week	36w	21.9 ± 7.0	
		CON	38 (22/18)	62 ± 7.61	CON	/		21.0 ± 6.3	
Mandal 2018 (Mandal et al., 2018)	UK	ARTR	17 (4/13)	57.8 ± 12.7	SC+RP	/	12w		
		CON	20 (7/13)	61.4 ± 12.9	NIV	≥4h, 7days/week			
Servantes 2012 (Servantes et al., 2012)	Brazil	ARTR	17 (8/9)	50.82 ± 9.45	AT+ST	50-65min+12–16 groups, 3-4days/week	12w	26.4 ± 17.6	
		ATR	17 (8/9)	51.76 ± 9.83	AT	50-65min, 3-4days/week		25.2 ± 24.7	
		CON	11 (5/6)	53.00 ± 8.19	CON	/		22.8 ± 17.4	
Mendelson 2016 (Mendelson et al., 2016)	Canada	ATR	17 (16/1)	63.8 ± 8.0	AT	50min, 5days/week	4w	31.1 ± 12.9	
		CON	17 (14/3)	59.6 ± 11.8	CON	/		28.1 ± 13.5	
Sengul 2011 (Sengul et al., 2011)	Turkey	ATR	10 (10/0)	54.40 ± 6.57	RE+AT	1.5h, 3days/week	12w	15.19 ± 5.43	8.20 ± 6.14
		CON	10 (10/0)	48.0 ± 7.49	CON	/		17.92 ± 6.45	3.42 ± 5.07
Garcia 2020 (Jurado-García et al., 2020)	Spain	ATR	34 (20/14)	52 ± 6.6	PUWP	/	24w	29 ± 19.7	9 ± 4.5
		CON	34 (23/11)	50 ± 9.5	BER	/		27 ± 10.4	10 ± 4.3
Foster 2009 (Foster et al., 2009)	USA	ATR	125 (48/77)	61.2 ± 6.6	Intensive Lifestyle Intervention	25min, 7times/week	48w	22.9 ± 18.0	
		CON	139 (60/79)	61.3 ± 6.4	Diabetes Support and Education	/		23.5 ± 15.0	
Guimaraes 2009 (Georgoulis et al., 2021)	Brazil	RMR	16 (10/5)	51.5 ± 6.8	OPE	30min, 7ays/week	12w	22.4 ± 4.8	14 ± 5
		CON	15 (11/40)	47.7 ± 9.8	NDP	30min, 7ays/week		22.4 ± 5.4	14 ± 7
Vranish 2016 (Vranish and Bailey, 2016)	USA	RMR	12	61.5 ± 3.9	IMT	150min, 7days/week	6w	22.2 ± 4.5	
		CON	12	69.1 ± 3.4	sham IMT	150min, 7days/week		32.3 ± 10.7	
Ishiyama 2017 (Ishiyama et al., 2017)	Japan	RMR	13 (11/2)	52.8 ± 10.1	MOM	45min, 7days/week	4w	21.3 ± 6.0	11.1 ± 3.6
		CON	12 (10/2)	49.9 ± 9.5	PNE	45min, 7days/week		21.7 ± 13.4	10.0 ± 4.8
Kuo 2017 (Kuo et al., 2017)	Taiwan, China	RMR	13 (11/2)	44.3 ± 2.9	EMST	25times/day, 5days/week	5w	16.5 ± 2.2	9.9 ± 1.1
		CON	12 (10/2)	48.0 ± 3.1	sham EMST	25times/day, 5days/week		14.6 ± 1.5	9.8 ± 0.9
Souza 2018 (Souza et al., 2018)	Brazil	RMR	8 (4/4)	54.8 ± 6.9	IMT	30min, 7times/week	12w	54.2 ± 27.4	12.9 ± 4.7
		CON	8 (6/2)	49.9 ± 11.6	P-IMT	30min, 7times/week		55.4 ± 28.9	10.8 + 6.4
Atilgan 2022 (Atilgan et al., 2020)	Turkey	RMR	14	53.71 ± 7.08	OE	30min, 5times/week	12w	27.6 ± 11.9	11.1 ± 4.5
		RMR	15	49.66 ± 9.08	IMT	30min, 7times/week		34.0 ± 18.4	11.1 ± 6.8
		CON	12	47.25 ± 7.32	Control	/			
Moawd 2020 (Moawd et al., 2020)	Egypt	RMR	28 (20/8)	55.5 ± 9.8	IMT	30min, 3times/week	12w	42.6 ± 27.1	8.14 ± 6.27
		CON	27 (22/5)	59.5 ± 4.8	P-IMT	30min, 3times/week		30.00 ± 19.33	8.93 ± 4.41
Azeredo 2022 (Azeredo et al., 2022)	Brazil	RMRCWPAP	8 (4/4)	67 ± 13	Inspiratory muscle training- CPAP+	≥4h, 7days/week	12w	53 ± 23.70	14 ± 7.41
		RMR	22 (13/9)	63 ± 15	Inspiratory muscle training- CPAP–	≥4h, 7days/week		29 ± 9.63	8 ± 4.44
		PAP	14 (7/7)	55 ± 15	Control- CPAP+	≥4h, 7days/week		44 ± 45.93	9 ± 5.93
		CON	21 (14/7)	55 ± 15	Control- CPAP–	≥4h, 7days/week		20 ± 10.37	11 ± 4.44
Poncin 2022 (Poncin et al., 2022)	Switzerland	RMR	12 (8/4)	48.0 ± 7.93	TE	15min, 4days/week	6w	18.9 ± 20.15	10.0 ± 8
		CON	13 (6/7)	56.0 ± 8.15	CON	3times/day, 4days/week		16.8 ± 16.30	8.0 ± 4.81
Qian 2022 (Qian et al., 2022)	China	RMR	30 (22/8)	59.13 ± 9.41	OMT	80min, 12times/week	4w	27.36 ± 7.23	
		CON	30 (25/5)	61.20 ± 10.68	TSC	20min, 12times/week		26.07 ± 6.01	
Leao 2024 (Cavalcante-Leao et al., 2024)	Brazil	RMR (inspiratory muscle)	5	25.0 ± 4.84	IMT	30times/day, 5days/week	12w	13.54 ± 6.7	
		RMR (expiratory muscle)	5	35.4 ± 15.35	PEP	30times/day, 5days/week		11.04 ± 5.78	
		CON	5	27.6 ± 5.12	CON	30times/day, 5days/week		7.48 ± 4.44	
Marzouqah 2024 (Marzouqah et al., 2024)	Canada	RMR	15 (11/4)	66.7 ± 11.4	OPE	30min, 10times/week	6w	24.8 ± 8.0	
		CON	15 (8/7)	68.0 ± 11.8	Sham OPE	30min, 10times/week		17.8 ± 9.4	
Reina 2020 (O’Connor-Reina et al., 2020)	Spain	RMR	18 (14/4)	59.17 ± 8.07	AirwayGym	20min, 7times/week	12w	44.77 ± 16.18	10.47 ± 2.61
		CON	10 (8/2)	63.9 ± 11.10	Control	/		47.36 ± 12.99	9.3 ± 4
Sankari 2024 (Sankari et al., 2024)	USA	RMR	12 (12/0)	66.3 ± 10.0	OPT+RMT	30min, 7times/week	12w	49.9 ± 22.3	4.8 ± 5.0
		CON	12 (10/2)	64.8 ± 7.7	CON	30min, 7times/week		44.2 ± 24.6	5.9 ± 2.3
Siripajana 2024 (Siripajana et al., 2024)	Thailand	RMR	12 (5/7)	54.58 ± 12.13	OE	10min, 4times/week	8w		7.833 ± 3.35
		CON	11 (4/7)	55.36 ± 12.92	Sham	10min, 4times/week			6.73 ± 3.93
Lin 2020 (Lin et al., 2020)	Taiwan, China	RMR	8 (5/3)	49.5 9.7	Intervention	80min, 2times/week	12w	47.0 ± 19.4	
		CON	7 (5/2)	52.7 4.6	CON	/		35.8 ± 17.5	
Paolucci 2023 (Paolucci et al., 2023)	Italy	RMR	28 (13/15)	61.46 ± 8.74	OMF+MRT+SHG	90min, 6times/week	6 ± 2w	8.35 ± 7.26	6.5 ± 10.37
		CON	24 (9/15)	60.42 ± 6.61	OMF+SHG	60min, 6times/week		9.25 ± 6.15	7.5 ± 8.89
Ieto 2015 (Ieto et al., 2015)	Brazil	RMR	19 (8/11)	48 ± 14	NI+STOM	8min, 21times/week	12w	15.6 ± 9.3	7.0 ± 5.93
		CON	20 (9/11)	45 ± 13	ND+NI+BBT	15min, 21times/week		15.1 ± 9.5	9.0 ± 4.81
Puhan 2006 (Puhan et al., 2006)	Switzerland	RMR	14 (12/2)	49.9 (6.7	Didgeridoo	20min, 5times/week	16w	22.3 ± 5.0	11.8 ± 3.5
		CON	11 (9/2)	47.0 (8.9	CON	/		19.9 ± 4.7	11.1 ± 6.4
Villa 2017 (Villa et al., 2017)	Italy	RMR	36 (14/22)	67 ± 2.3	OPT+ND	7times/week	6w		
		CON	18 (8/10)	65 ± 2.8	ND	7times/week	6w		

### 3.3 Risk of bias assessment

**Figure 2:**
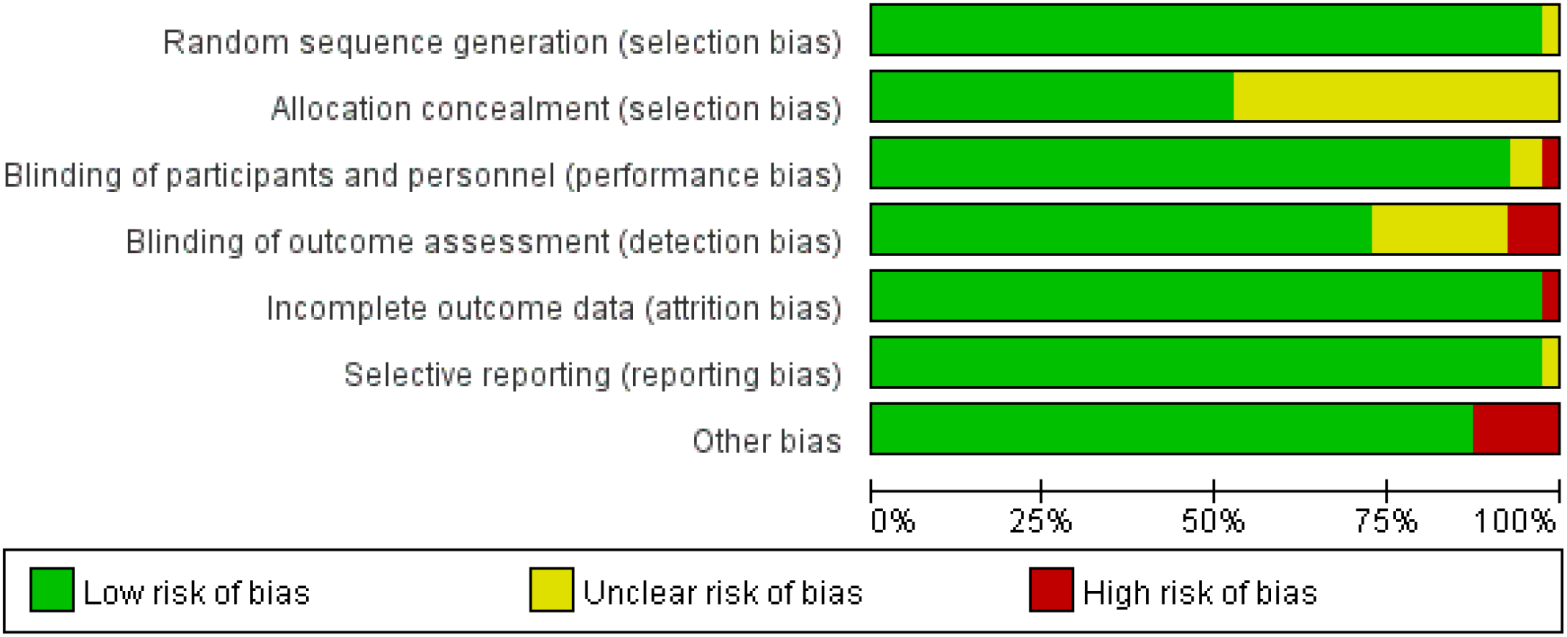
Risk of bias in studies included in this meta-analysis.

**Figure 3:**
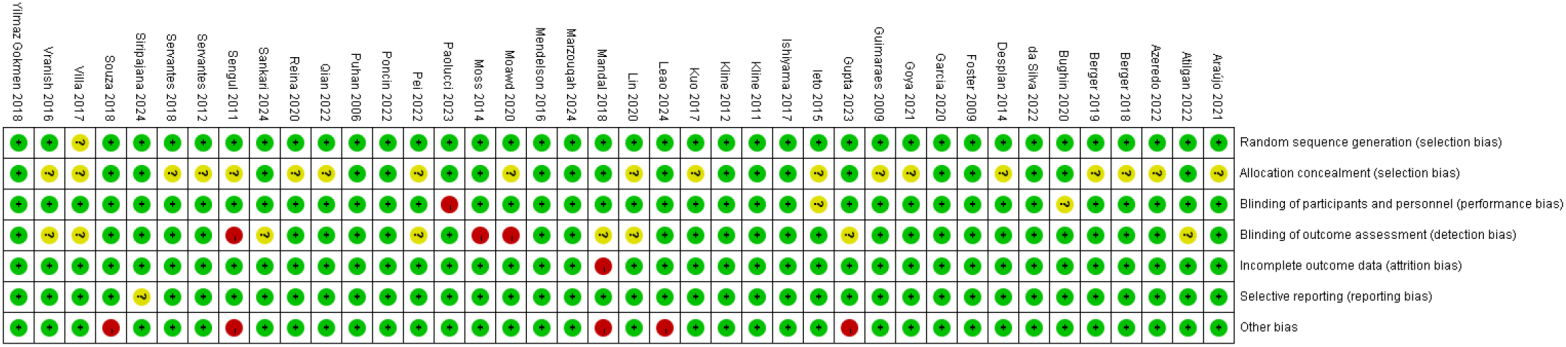
Evaluation results for each methodological quality item included in the study.

Based on the Cochrane ROB 2.0 tool assessment, studies included in this systematic review generally demonstrated good methodological quality regarding random sequence generation, with the vast majority rated as low risk. Regarding allocation concealment and blinding of outcome assessors, approximately half of the studies provided adequate methodological descriptions and met the low-risk criteria, while the remaining studies were rated as uncertain risk due to unclear reporting of information. Owing to the specific nature of rehabilitation interventions, most studies encountered difficulties in implementing blinding for both patients and therapists, resulting in a generally elevated risk of bias across this field. Regarding data integrity, the majority of studies were rated as low risk for incomplete outcome data and selective reporting of results. Notably, individual studies were rated as high risk due to baseline imbalance between groups. Overall, the risk of bias in the included studies was acceptable, predominantly low or moderate risk. **Table 2**, **Figures 2**, **3**.

**Table 2:** Risk of bias assessment results for included studies.

Study	Random sequence generation	Allocation concealment	Blinding ofparticipants and personnel	Blinding of outcome assessment	Incomplete outcome data	Selective reporting	Other bias
Araujo 2021 (Araújo et al., 2021)	L	U	L	L	L	L	L
Atilgan 2022 (Atilgan et al., 2020)	L	L	L	U	L	L	H
Azeredo 2022 (Azeredo et al., 2022)	L	U	L	L	L	L	L
Berger 2018 (Berger et al., 2018)	L	U	L	L	L	L	L
Berger 2019 (Berger et al., 2019)	L	U	L	L	L	L	L
Bughin 2020 (Bughin et al., 2020)	L	L	U	L	L	L	L
Da Silva 2022 (Da Silva et al., 2022)	L	L	L	L	L	L	L
Desplan 2014 (Desplan et al., 2014)	L	U	L	L	L	L	H
Foster 2009 (Foster et al., 2009)	L	L	L	L	L	L	L
Garcia 2020 (Jurado-García et al., 2020)	L	L	L	L	L	L	L
Goya 2021 (Goya et al., 2021)	L	U	L	L	L	L	L
Guimaraes 2009 (Georgoulis et al., 2021)	L	U	L	H	H	L	L
Gupta 2023 (Gupta et al., 2023)	L	L	L	U	L	L	H
Ieto 2015 (Ieto et al., 2015)	L	U	U	L	L	L	L
Ishiyama 2017 (Ishiyama et al., 2017)	L	L	L	L	L	L	L
Kline 2011 (Kline et al., 2011)	L	L	L	L	L	L	L
Kline 2012 (Kline et al., 2012)	L	L	L	L	L	L	L
Kuo 2017 (Kuo et al., 2017)	L	U	L	L	L	L	L
Leao 2024 (Cavalcante-Leao et al., 2024)	L	L	L	L	L	L	H
Lin 2020 (Lin et al., 2020)	L	U	L	U	L	L	H
Mandal 2018 (Mandal et al., 2018)	L	L	L	U	H	L	H
Marzouqah 2024 (Marzouqah et al., 2024)	L	L	L	L	L	L	L
Mendelson 2016 (Mendelson et al., 2016)	L	L	L	L	L	L	L
Moawd 2020 (Moawd et al., 2020)	L	U	L	H	L	L	L
Moss 2014 (Moss et al., 2014)	L	L	L	H	L	L	L
Paolucci 2023 (Paolucci et al., 2023)	L	L	H	L	L	L	L
Pei 2022 (Pei et al., 2022)	L	U	L	U	L	L	L
Poncin 2022 (Poncin et al., 2022)	L	L	L	L	L	L	L
Puhan 2006 (Puhan et al., 2006)	L	L	L	L	L	L	L
Qian 2022 (Qian et al., 2022)	L	U	L	L	L	L	L
Reina 2020 (O’Connor-Reina et al., 2020)	L	U	L	L	L	L	L
Sankari 2024 (Sankari et al., 2024)	L	L	L	U	L	L	L
Sengul 2011 (Sengul et al., 2011)	L	U	L	H	L	L	H
Servantes 2012 (Servantes et al., 2012)	L	U	L	L	L	L	L
Servantes 2018 (Servantes et al., 2018)	L	U	L	L	L	L	L
Siripajana 2024 (Siripajana et al., 2024)	L	L	L	L	L	U	L
Souza 2018 (Souza et al., 2018)	L	L	L	L	L	L	H
Villa 2017 (Villa et al., 2017)	U	U	L	U	L	L	L
Vranish 2016 (Vranish and Bailey, 2016)	L	U	L	U	L	L	L
Yilmaz Gokmen 2018 (Yilmaz Gokmen et al., 2019)	L	L	L	L	L	L	L

### 3.4 Direct pairwise meta-analysis

Compared with CON, MBE demonstrated significant efficacy in improving SI (SMD = −1.34, 95% CI [−2.44, −0.23], P = 0.02), though substantial heterogeneity existed between studies (I² = 87%), indicating considerable variation in intervention effects across research. HIIT yielded markedly superior improvements (SMD = −4.07, 95% CI [−5.49, −2.65], P < 0.00001), with relatively manageable inter-study heterogeneity (I² = 67%); CT also demonstrated significant efficacy (SMD = -1.33, 95% CI [-2.21, -0.44], P = 0.003), though with high inter-study heterogeneity (I² = 86%); AT showed no statistically significant advantage (SMD = -1.90, 95% CI [-4.44, 0.64], P = 0.14), with extremely high heterogeneity between studies (I² = 99%); RE demonstrated a clear improvement effect (SMD = -0.60, 95% CI [-1.10, -0.10], P = 0.02), though with considerable heterogeneity between studies (I² = 87%); ACRT demonstrated significant efficacy (SMD = -1.67, 95% CI [-2.50, -0.84], P < 0.0001), though marked heterogeneity persisted (I² = 89%). Direct comparisons revealed no significant difference between AT and ACRT in improving SI (SMD = -0.11, 95% CI [-0.78, 0.56], P = 0.74); However, RE demonstrated a significant disadvantage compared with ACRT (SMD = −2.97, 95% CI [−4.11, −1.82], P < 0.0001). This finding provides important reference for clinical selection of intervention programmed. **Appendix 1**.

Compared with CON, MBE demonstrated a marginally significant effect on improving CE (SMD = 0.97, 95% CI [-0.02, 1.95], P = 0.05), though with considerable heterogeneity across studies (I² = 79%); HIIT demonstrated markedly superior improvements (SMD = 3.06, 95% CI [2.06, 4.05], P < 0.00001); CT also demonstrated significant efficacy (SMD = 2.17, 95% CI [0.98, 3.35], P = 0.0003), though with high inter-study heterogeneity (I² = 90%); AT failed to achieve statistical significance (SMD = 0.76, 95% CI [-0.31, 1.83], P = 0.16), with substantial heterogeneity between studies (I² = 88%); RE demonstrated a clear improvement effect (SMD = 0.92, 95% CI [0.36, 1.48], P = 0.001), though with marked heterogeneity between studies (I² = 85%); ACRT demonstrated significant efficacy (SMD = 1.59, 95% CI [0.81, 2.38], P < 0.0001), though with high inter-study heterogeneity (I² = 90%). Direct comparisons revealed no significant difference between AT and ACRT in improving CE (SMD = 0.04, 95% CI [-0.64, 0.71], P = 0.92). **Appendix 1**.

Compared with CON, MBE (SMD = −0.20, 95% CI [−0.85, 0.45], P = 0.54) and RE (SMD = −0.15, 95% CI [−0.42, 0.00], P = 0.54) showed no significant difference in BC 95% CI [-0.85, 0.45], P = 0.54) and RE (SMD = -0.15, 95% CI [-0.42, 0.12], P = 0.28, I² = 0%) demonstrated no significant therapeutic effect. HIIT (SMD = −0.96, 95% CI [−2.15, 0.22], P = 0.11, I² = 90%) and CT (SMD = −0.72, 95% CI [-1.59, 0.14], P = 0.10, I² = 87%) and AT (SMD = -3.02, 95% CI [-7.39, 1.35], P = 0.18, I² = 99%) also failed to achieve statistical significance, with high heterogeneity present across all three studies. Only ACRT demonstrated a significant improvement effect (SMD = -0.44, 95% CI [-0.82, -0.06], P = 0.02, I² = 58%). Direct comparisons revealed no significant difference between RE and ACRT in improving BC (SMD = 0.00, 95% CI [-0.81, 0.81], P = 1.00). **Appendix 1** .

### 3.5 Network meta-analysis

#### 3.5.1 Network diagrams included in the study

**Figure 4:**
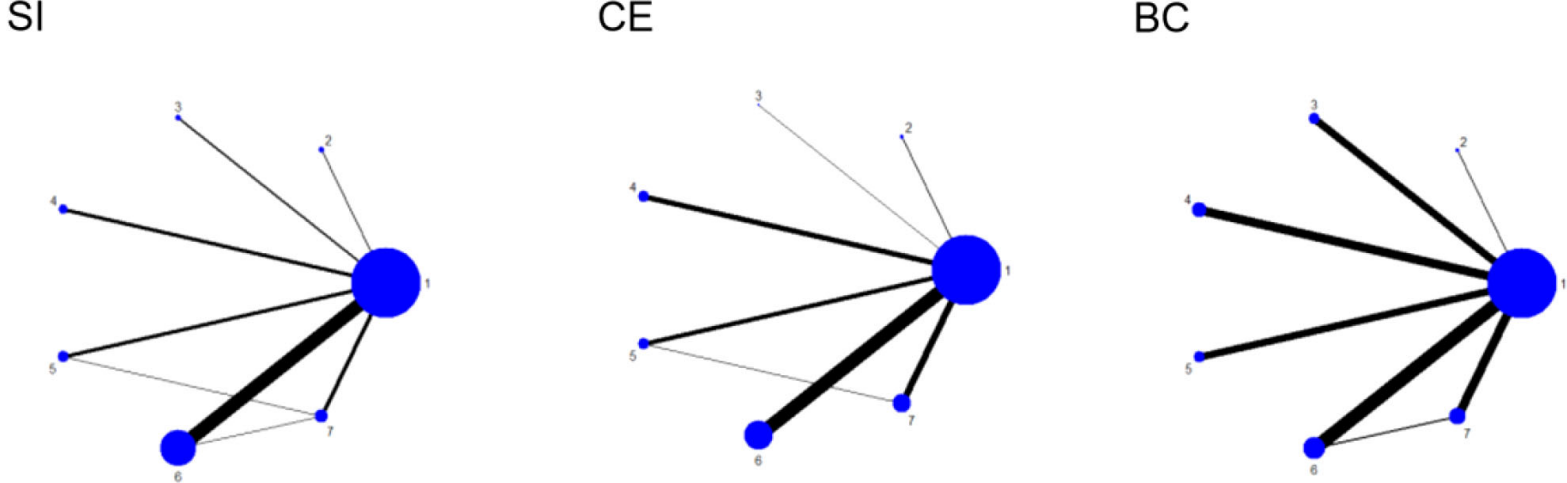
Network plot of outcome indicators. 1, CON; 2, MBE; 3, HIIT; 4, CT; 5, AT; 6, RE; 7, ACRT.

The seven nodes in the diagram represent seven intervention measures, with straight lines between nodes indicating direct comparisons between interventions. The thickness of the lines denotes the number of studies comparing the two interventions directly. Among these, RE is the most extensively studied intervention, whereas research on MBE is relatively scarce. The network diagram for outcome measures is detailed in **Figure 4**.

#### 3.5.2 Ranking of intervention effects across six different exercise modalities

**Figure 5:**
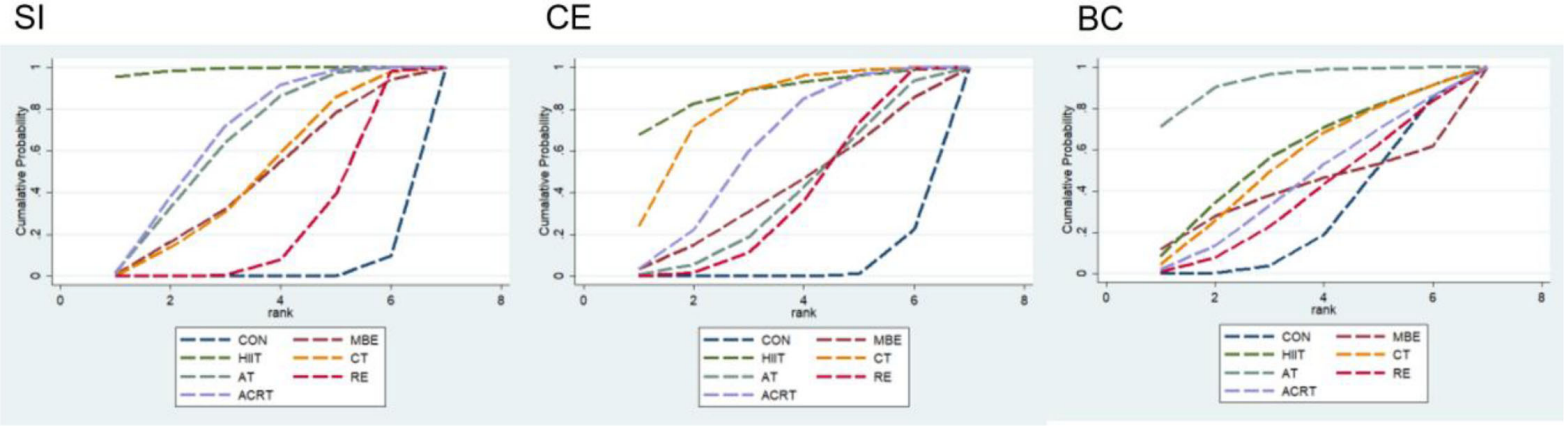
Effectiveness ranking chart for outcome measures.

The efficacy ranking of seven interventions in improving SI among SAS patients was as follows: HIIT (SUCRA = 98.9) > ACRT (SUCRA = 67.0) > AT (SUCRA = 64.0) > CT (SUCRA = 47.2) > MBE (SUCRA = 46.9) > RE (SUCRA = 24.4) > CON (SUCRA = 1.7). High-intensity interval training (HIIT) demonstrated the highest probability of being the optimal intervention (PrBest=95.1%), significantly surpassing other interventions, while the control group (CON) yielded the weakest effect (PrBest=0.0%). **Table 3**, **Figure 5**.

**Table 3:** Ranking of six exercise interventions for improving SAS patients.

Treatment	SI		CE		BC	
	SUCRA(%)	Rank	SUCRA(%)	Rank	SUCRA(%)	Rank
CON	1.7	7	4.0	7	26.8	7
MBE	46.9	5	41.2	4	39.9	5
HIIT	98.9	1	87.9	1	57.2	2
CT	47.2	4	79.9	2	53.4	3
AT	64.0	3	38.6	5	92.7	1
RE	24.4	6	37.1	6	36.9	6
ACRT	67.0	2	61.3	3	43.0	4

The seven interventions ranked in order of effectiveness for improving CE in SAS patients were: HIIT (SUCRA = 87.9) > CT (SUCRA = 79.9) > ACRT (SUCRA = 61.3) > MBE (SUCRA = 41.2) > AT (SUCRA = 38.6) > RE (SUCRA = 37.1) > CON (SUCRA = 4.0). High-intensity interval training (HIIT) exhibited the highest probability of being the optimal intervention (PrBest=67.8%), with cycle training (CT) being the second-best intervention at 23.9%, while the control group (CON) demonstrated the weakest efficacy (PrBest=0.0%). **Table 3**, **Figure 5**.

The seven interventions ranked in order of effectiveness for improving BC in SAS patients were: AT (SUCRA = 92.7) > HIIT (SUCRA = 57.2) > CT (SUCRA = 53.4) > ACRT (SUCRA = 43.0) > MBE (SUCRA = 39.9) > RE (SUCRA = 36.9) > CON (SUCRA = 26.8). Among these, aerobic training (AT) exhibited the highest probability of being the optimal intervention (PrBest=71.4%), while high-intensity interval training (HIIT) had an 8.6% probability of being the second-best intervention. The control group (CON) demonstrated the weakest relative efficacy (PrBest=0.0%). **Table 3**, **Figure 5**.

#### 3.5.3 Estimation of pooled effect sizes for primary outcome measures

Compared with CON, HIIT (SMD = -4.07, 95% CI [-5.49, -2.65]), ACRT (SMD = -1.67, 95% CI [-2.50, -0.84]), AT (SMD = −1.90, 95% CI [-4.44, 0.64]), CT (SMD = -1.33, 95% CI [-2.21, -0.44]), MBE (SMD = −1.34, 95% CI [−2.44, −0.23]), and RE (SMD = −0.60, 95% CI [−1.10, −0.10]) all demonstrated varying degrees of positive effects in improving SI among SAS patients. The ranked efficacy of different exercise therapies in improving SI was: HIIT (SUCRA = 98.9) > ACRT (SUCRA = 67.0) > AT (SUCRA = 64.0) > CT (SUCRA = 47.2) > MBE (SUCRA = 46.9) > RE (SUCRA = 24.4) > CON (SUCRA = 1.7). **Table 4**.

**Table 4:** Network meta-analysis matrix for outcome measures.

SI						
HIIT	2.17 (0.03,4.30)	2.24 (0.02,4.45)	2.75 (0.47,5.03)	2.79 (0.27,5.30)	3.45 (1.49,5.41)	4.14 (2.29,5.99)
-2.17 (-4.30,-0.03)	ACRT	0.07 (-1.43,1.56)	0.58 (-1.13,2.29)	0.62 (-1.39,2.63)	1.28 (0.07,2.49)	1.97 (0.91,3.04)
-2.24 (-4.45,-0.02)	-0.07 (-1.56,1.43)	AT	0.51 (-1.29,2.32)	0.55 (-1.54,2.64)	1.21 (-0.16,2.58)	1.90 (0.69,3.12)
-2.75 (-5.03,-0.47)	-0.58 (-2.29,1.13)	-0.51 (-2.32,1.29)	CT	0.04 (-2.12,2.20)	0.70 (-0.78,2.18)	1.39 (0.06,2.72)
-2.79 (-5.30,-0.27)	-0.62 (-2.63,1.39)	-0.55 (-2.64,1.54)	-0.04 (-2.20,2.12)	MBE	0.66 (-1.16,2.48)	1.35 (-0.35,3.06)
-3.45 (-5.41,-1.49)	-1.28 (-2.49,-0.07)	-1.21 (-2.58,0.16)	-0.70 (-2.18,0.78)	-0.66 (-2.48,1.16)	RE	0.69 (0.04,1.34)
-4.14 (-5.99,-2.29)	-1.97 (-3.04,-0.91)	-1.90 (-3.12,-0.69)	-1.39 (-2.72,-0.06)	-1.35 (-3.06,0.35)	-0.69 (-1.34,-0.04)	CON
CE						
HIIT	-0.83 (-3.89,2.23)	-1.51 (-4.45,1.43)	-2.07 (-5.43,1.29)	-2.11 (-5.15,0.93)	-2.13 (-5.01,0.74)	-3.06 (-5.83,-0.28)
0.83 (-2.23,3.89)	CT	-0.68 (-2.29,0.93)	-1.24 (-3.53,1.04)	-1.28 (-3.06,0.49)	-1.30 (-2.80,0.19)	-2.22 (-3.51,-0.94)
1.51 (-1.43,4.45)	0.68 (-0.93,2.29)	ACRT	-0.56 (-2.69,1.56)	-0.60 (-2.04,0.83)	-0.62 (-1.85,0.60)	-1.55 (-2.52,-0.58)
2.07 (-1.29,5.43)	1.24 (-1.04,3.53)	0.56 (-1.56,2.69)	MBE	-0.04 (-2.30,2.21)	-0.06 (-2.10,1.97)	-0.98 (-2.87,0.91)
2.11 (-0.93,5.15)	1.28 (-0.49,3.06)	0.60 (-0.83,2.04)	0.04 (-2.21,2.30)	AT	-0.02 (-1.46,1.42)	-0.94 (-2.17,0.29)
2.13 (-0.74,5.01)	1.30 (-0.19,2.80)	0.62 (-0.60,1.85)	0.06 (-1.97,2.10)	0.02 (-1.42,1.46)	RE	-0.92 (-1.68,-0.17)
3.06 (0.28,5.83)	2.22 (0.94,3.51)	1.55 (0.58,2.52)	0.98 (-0.91,2.87)	0.94 (-0.29,2.17)	0.92 (0.17,1.68)	CON
BC						
AT	1.98 (-1.27,5.24)	2.18 (-0.91,5.27)	2.53 (-0.45,5.51)	2.77 (-2.35,7.90)	2.78 (-0.05,5.61)	2.97 (0.67,5.28)
-1.98 (-5.24,1.27)	HIIT	0.20 (-2.89,3.29)	0.55 (-2.43,3.53)	0.79 (-4.33,5.92)	0.80 (-2.03,3.62)	0.99 (-1.31,3.29)
-2.18 (-5.27,0.91)	-0.20 (-3.29,2.89)	CT	0.35 (-2.44,3.14)	0.59 (-4.43,5.61)	0.60 (-2.04,3.23)	0.79 (-1.26,2.85)
-2.53 (-5.51,0.45)	-0.55 (-3.53,2.43)	-0.35 (-3.14,2.44)	ACRT	0.24 (-4.71,5.20)	0.25 (-2.07,2.56)	0.44 (-1.45,2.33)
-2.77 (-7.90,2.35)	-0.79 (-5.92,4.33)	-0.59 (-5.61,4.43)	-0.24 (-5.20,4.71)	MBE	0.00 (-4.86,4.87)	0.20 (-4.38,4.78)
-2.78 (-5.61,0.05)	-0.80 (-3.62,2.03)	-0.60 (-3.23,2.04)	-0.25 (-2.56,2.07)	-0.00 (-4.87,4.86)	RE	0.20 (-1.45,1.84)
-2.97 (-5.28,-0.67)	-0.99 (-3.29,1.31)	-0.79 (-2.85,1.26)	-0.44 (-2.33,1.45)	-0.20 (-4.78,4.38)	-0.20 (-1.84,1.45)	CON

Compared with CON, HIIT (SMD = 3.06, 95% CI [2.06, 4.05]), CT (SMD = 2.17, 95% CI [0.98, 3.35]), ACRT (SMD = 1.59, 95% CI [0.81, 2.38]), MBE (SMD = 0.97, 95% CI [-0.02, 1.95]), AT (SMD = 0.76, 95% CI [-0.31, 1.83]), and RE (SMD = 0.92, 95% CI [0.36, 1.48]) all demonstrated positive trends in improving CE among SAS patients. The ranked efficacy of different exercise therapies for improving CE was: HIIT (SUCRA = 87.9) > CT (SUCRA = 79.9) > ACRT (SUCRA = 61.3) > MBE (SUCRA = 41.2) > AT (SUCRA = 38.6) > RE (SUCRA = 37.1) > CON (SUCRA = 4.0). **Table 4**.

Compared with CON, only ACRT (SMD = −0.44, 95% CI [−0.82, −0.06]) demonstrated a significant effect in improving BC among SAS patients. The ranked efficacy of different exercise therapies in improving BC was: AT (SUCRA = 92.7) > HIIT (SUCRA = 57.2) > CT (SUCRA = 53.4) > ACRT (SUCRA = 43.0) > MBE (SUCRA = 39.9) > RE (SUCRA = 36.9) > CON (SUCRA = 26.8). **Table 4**.

### 3.6 Small-sample effects or publication bias tests

**Figure 6:**
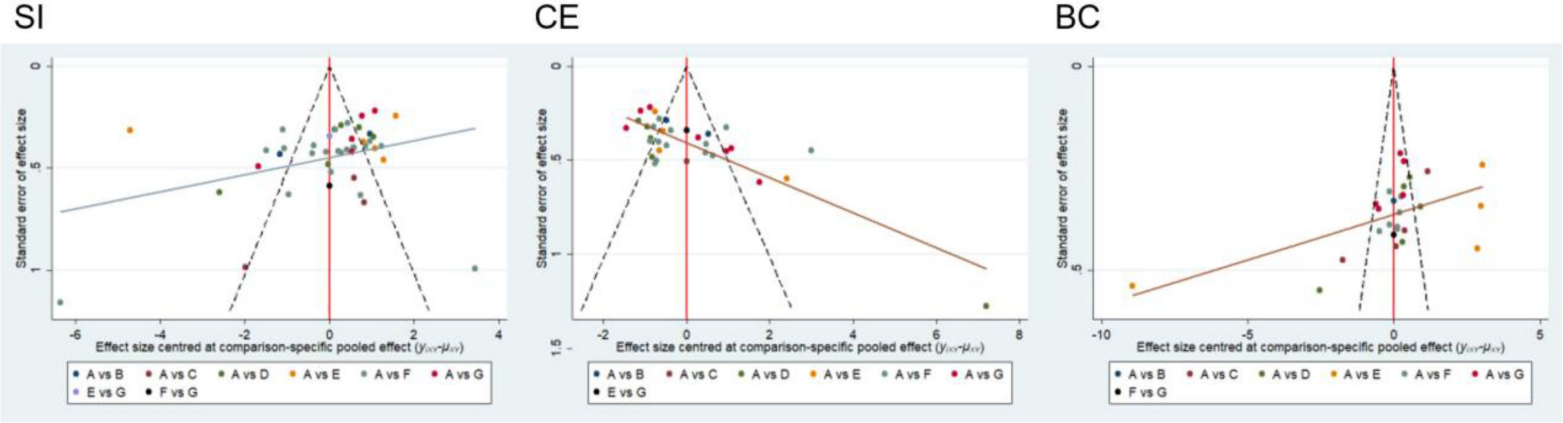
Corrected comparison funnel plot for outcome indicators. A, CON; B, MBE; C, HIIT; D, CT; E, AT; F, RE; G, ACRT.

A corrected funnel plot was employed to assess small-sample effects and examine publication bias in studies included in the network meta-analysis. Results indicated that the funnel plots for the included studies were largely symmetrical, suggesting no small-sample effects were present in this research. Furthermore, no significant publication bias was detected, as detailed in **Figure 6**.

## 4 Discussion

This network meta-analysis systematically compared the effects of six exercise therapies on key outcome measures in patients with sleep apnoea syndrome. The findings revealed distinct therapeutic characteristics across three core outcome domains, providing crucial evidence for individualised treatment approaches.

Regarding SI, HIIT demonstrated the most favourable intervention effects, with its standardised mean difference significantly outperforming the control group. It ranked first in both SUCRA ranking (98.9) and probability of being the best intervention (95.1%). This finding aligns closely with prior research, indicating that high-intensity interval training, through its unique exercise intensity pattern, can rapidly improve autonomic nervous system regulation, enhance parasympathetic tone, and potentially redistribute fluid from the cervical region, thereby effectively reducing upper airway collapse during sleep (Lins-Filho et al., 2024). Although AT demonstrated a trend towards improvement in point estimates, it failed to achieve statistical significance relative to CON and ranked markedly below HIIT in direct comparisons. This suggests that exercise intensity, rather than merely volume or duration, may be a key determinant in improving sleep-related respiratory parameters in SAS patients (Lins-Filho et al., 2020). ACRT and CT likewise exhibited significant benefits, supporting the role of combined or diversified training protocols in this field.

Regarding CE, HIIT was again rated as the optimal intervention (SUCRA = 87.9, PrBest=67.8%), with a highly significant effect size (SMD = 3.06). This aligns closely with findings from extensive studies in other populations, confirming HIIT’s exceptional efficacy in enhancing peak oxygen uptake. This may stem from its potent stimulation of cardiovascular and mitochondrial adaptations (Weston et al., 2014). Notably, CT ranked second (SUCRA = 79.9), outperforming ACRT. This may be attributed to the continuous nature of circuit training, which typically maintains higher average heart rates than traditional isolated aerobic and resistance training, thereby providing a more potent combined stimulus for cardiovascular improvement (Schroeder et al., 2019). The effects of AT and RE on enhancing CE were relatively limited, potentially linked to specific intervention protocols (such as exercise intensity in AT) or the fact that RE primarily targets respiratory muscles rather than central cardiovascular function (Vranish and Bailey, 2016).

Regarding improvements in BC, studies exhibited divergent patterns. Aerobic training (AT) was rated as the most effective intervention (SUCRA = 92.7, PrBest=71.4%), although its pooled SMD compared to the control group failed to reach statistical significance due to extremely high heterogeneity. This heterogeneity likely stemmed from substantial variations in AT protocols (type, duration, frequency) across different studies (Kline et al., 2011). Nevertheless, its high-ranking outcome aligns with aerobic exercise’s established role in promoting fat oxidation and reducing visceral adipose tissue (a key pathological factor in SAS) (Blackman et al., 2016). Notably, ACRT was the sole intervention demonstrating statistically significant superiority in BC improvement (SMD = −0.44, p = 0.02 compared to CON). This suggests that incorporating resistance training may offer more consistent (albeit modest) benefits in improving BC compared to aerobic training alone (Bateman et al., 2011). HIIT and CT demonstrated relatively weaker performance in this domain, indicating that despite their superior efficacy in enhancing cardiorespiratory fitness and sleep quality, their time-efficient nature may not have generated sufficient energy deficits within the existing study protocols to achieve fat reduction comparable to longer duration aerobic training (Wewege et al., 2017).

The core finding of this study lies in the differential ranking of various interventions across outcome measures. HIIT appears to be the most comprehensive single exercise modality, demonstrating optimal efficacy in improving SI and CE, while also showing benefits in enhancing BC. This renders it an efficient choice for simultaneously addressing multiple functional impairments associated with sleep-related breathing disorders (Vivodtzev et al., 2018). For patients prioritising body fat reduction as their primary therapeutic goal, aerobic training (AT) may warrant consideration as the preferred intervention. ACRT offers a balanced therapeutic strategy, demonstrating significant (though not optimal) benefits across all three domains, potentially facilitating long-term adherence and overall health promotion (Peng et al., 2022). RE and MBE rank comparatively lower, suggesting they are better suited as adjunct therapies rather than first-line exercise interventions for SAS, although they retain value for specific subgroups or addressing issues such as stress and respiratory control (Andrea Ban et al., 2024).

### 4.1 Limitations

In our analysis, the corrected comparison funnel plot was used as the primary tool for detecting small-study effects, and it demonstrated general symmetry. To address your concern and enhance transparency, we have now explicitly acknowledged this limitation in the Discussion section (see below). Future updates of this meta-analysis, with a larger pool of studies, will allow for more robust quantitative assessments such as Egger’s test. Substantial heterogeneity was observed, particularly for body composition outcomes, likely arising from variations in patient characteristics (e.g., disease severity, age, BMI), exercise protocols (e.g., intensity, frequency, duration), and control group conditions across studies. Although a random-effects model was used to account for this variability, the pooled estimates should be interpreted with caution. Subgroup analyses to explore these sources were not feasible due to the limited number of studies per comparison, highlighting the need for future research with larger evidence bases. In addition, although this study included 40 randomized controlled trials, the duration of exercise interventions varied considerably across studies, with several trials implementing relatively short-term programs. Consequently, the long-term sustainability of the observed improvements in sleep indices, cardiopulmonary endurance, and body composition remains uncertain. Future large-scale randomized controlled trials with longer follow-up periods are required to determine whether the benefits of different exercise modalities can be maintained over time.

## 5 Conclusion

This network meta-analysis indicates that six exercise therapies produce differentiated improvements in sleep indices, cardiorespiratory endurance, and body composition among patients with sleep-related breathing disorders. HIIT demonstrated optimal efficacy in enhancing sleep indices and cardiorespiratory endurance, aerobic training showed the greatest potential for improving body composition, while aerobic-respiratory combined training provided relatively balanced overall benefits. In clinical practice, exercise intervention strategies should be selected individually based on patients’ specific conditions and primary treatment objectives. Future research should further clarify optimal exercise dosages, investigate long-term effects, and validate synergistic effects from combining different exercise modalities.

## Data Availability

The original contributions presented in the study are included in the article/**Supplementary Material**. Further inquiries can be directed to the corresponding author.
