# Supplementary figures and images for: Effects of six different exercise therapies on sleep indices, cardiopulmonary endurance, and body composition in patients with sleep-related breathing disorders: network meta-analysis of 40 RCTs
Source: Front Physiol. 2026 Mar 25;17:1773901. doi: 10.3389/fphys.2026.1773901 (PMC13056668; doi:10.3389/fphys.2026.1773901)

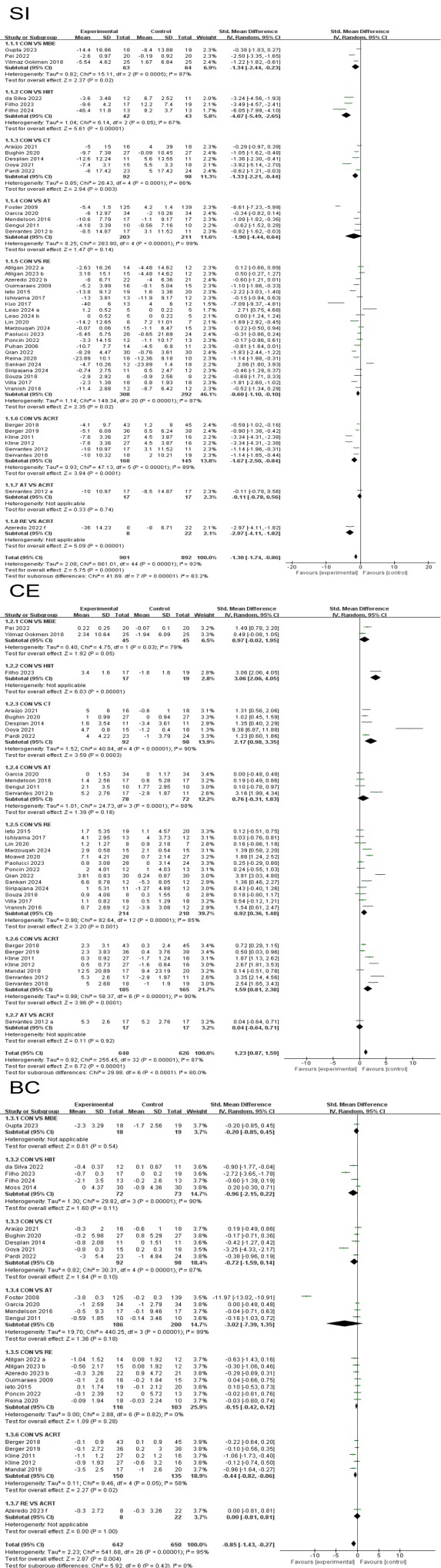


**Appendix 1** Forest plot of the outcomes

Supplement: Supplementary file 1 [file Table1.docx]
